# Supplementary material for: Effectiveness, acceptability, and feasibility of technology-enabled health interventions for adolescents living with HIV in low- and middle-income countries: A systematic review protocol
Source: PLoS One. 2023 Feb 17;18(2):e0281894. doi: 10.1371/journal.pone.0281894 (PMC9937495; doi:10.1371/journal.pone.0281894)
Supplement: S1 File — (DOCX) [file pone.0281894.s002.docx]

**Supporting information**

**S2: Data extraction form**

| **Reviewer** | **Article Number** | **Title of Article** |
| --- | --- | --- |
| **Authors** | **Journal, volume, issue** | **Year Published/Dates of Study** |
| **Country of Origin / Geographical location** | **Language** | **Study Objective/Aim** |
|  | **Intervention (Quantitative outcomes)** | **(Qualitative outcomes)** |
| **Study Population** |  |  |
| Number of Participants |  |  |
| Mean Age / age group |  |  |
| Gender |  |  |
| **Study Setting**  (where intervention provided e.g. home, clinic, community) |  |  |
| **Methods** | | |
| Design of study |  |  |
| Inclusion and exclusion criteria |  |  |
| Duration of follow-up |  |  |
| Recruitment methods and period |  |  |
| Completion rates |  |  |
| Sample size |  |  |
| **Intervention** | | |
| Description of intervention  ***(TIDieR) checklist*** |  |  |
| 1. *Brief name of health intervention* |  |  |
| 1. *Why (rationale, theory, goal)* |  |  |
| 1. *What (materials/content)* |  |  |
| 1. *What (procedures)* |  |  |
| 1. *Who provided (intervention agent)* |  |  |
| 1. *How – modes of delivery* |  |  |
| 1. *Where* |  |  |
| 1. *When and How much (Duration & intensity)* |  |  |
| **Use of technology** |  |  |
| 1. *Type of device (e.g., smartphone, computer, tablet)* |  |  |
| 1. *Technology design (e.g. interactive vs non-interactive, individuals vs groups, use of audio-visuals)* |  |  |
| 1. *Delivery platform / mode (e.g. application-based, web-based, SMS, phone support, social media)* |  |  |
| Comparison / control condition |  |  |
| **Outcomes** | | |
| Type of outcomes measured (e.g. patient reported, clinical, health care utilization) and unit of measure |  |  |
| Timing of measurements |  |  |
| Intention to treat analysis |  |  |
| Adverse outcomes |  |  |
| Acceptability |  |  |
| Feasibility |  |  |
| Usability / functionality / technological challenges |  |  |
| Perceived usefulness |  |  |
| **Results** | | |
| Summary data for each group and outcomes measured (means, sd, effect size) |  |  |
| Correspondence with authors required (Y/N) |  | |
