# Supplementary material for: Effectiveness, acceptability, and feasibility of technology-enabled health interventions for adolescents living with HIV in low- and middle-income countries: A systematic review protocol
Source: PLoS One. 2023 Feb 17;18(2):e0281894. doi: 10.1371/journal.pone.0281894 (PMC9937495; doi:10.1371/journal.pone.0281894)
Supplement: Attachment — Submitted filename: Response to Reviewers PLOS One.docx [file pone.0281894.s003.docx]

PONE-D-22-18587

Effectiveness, acceptability and feasibility of technology-enabled health interventions for adolescents living with HIV in low- and middle-income countries: a systematic review protocol

Dear Editor,

We thank the reviewers for their time and effort in improving the manuscript. Please see our responses to the reviewers’ comments below.

| **Reviewer/Editor comment** | **Response** | **Page** |
| --- | --- | --- |
| 1. Please ensure that your manuscript meets PLOS ONE's style requirements, including those for file naming. | We formatted the manuscript according to the style requirements. | N/A |
| 2. Thank you for stating the following financial disclosure:  "The funders had and will not have a role in study design, data collection and analysis, decision to publish, or preparation of the manuscript."  At this time, please address the following queries:  a) Please clarify the sources of funding (financial or material support) for your study. List the grants or organizations that supported your study, including funding received from your institution.  b) State what role the funders took in the study. If the funders had no role in your study, please state: “The funders had no role in study design, data collection and analysis, decision to publish, or preparation of the manuscript.”  c) If any authors received a salary from any of your funders, please state which authors and which funders.  d) If you did not receive any funding for this study, please state: “The authors received no specific funding for this work.”  Please include your amended statements within your cover letter; we will change the online submission form on your behalf. | We received no funding. The following statement was included: “The authors received no specific funding for this work.” | 12 |
| 3. Please include captions for your Supporting Information files at the end of your manuscript, and update any in-text citations to match accordingly. Please see our Supporting Information guidelines for more information: http://journals.plos.org/plosone/s/supporting-information.  [Note: HTML markup is below. Please do not edit.] | Caption included. S1 and S2 | Supplementary files. |
| Reviewer #1: This is a very well-written protocol study. Please consider the following:  1-Remove links from the manuscript and move them to the references section (e.g., Table 1, other considerations).  2-Change Box 1 to a table.  3-Add a discussion section considering the problem statements, objectives, and intended results of this study. | We thank the reviewer for the comments.  1- The link was removed (line 159) and a reference was added.  2 Box changed to table  3 Discussion section added. | 6  7  11 |
| Reviewer #2: Dear Author,  This is a research addressing a vulnerable population (adolescents living with HIV) in countries were prevalence is among the highest in the world, and for that this research is relevant in my opinion. The manuscript is well written. I think it is relevant to think in advance about how this review can be useful as it’s quite challenging. I suggest some comments that I hope can help you improve your review.  To give you some information about my background, I am an adolescent physician and HIV in adolescents is among my current research interest. I have not done any reviews myself I but have previously conducted an SMS intervention targeting adolescents with chronic condition and I have conducted qualitative individual interviews.  • I would remove the last 2 bullet points in the “Strengths and limitations’ part as it is expected that you use the adequate guidelines.  • About the definition of adolescents  In my understanding, you restrain the research to 10-19 years old adolescents, which can be ok. May I suggest you to consider to widen this age range to 10-24, as suggested by Susan Sawyer and al in their article “The age of adolescents”. Of course it’s a wide age range but it fits better this life phase and might be actually easier to include your articles, as we all know that it’s quite hard to find specific data on adolescents.  • First references  I do agree that you must highlight that this subgroup has lower adherence and retention in care, but the references 1 and 2 you use are in my opinion not adequate because their focus is mental health and not adherence or retention in care.  • Review questions  I find it very relevant to clearly formulate the review questions. To put it in simple words if I am correct: you would like to know what has been done and what works. From my short experience, I am afraid that as you point it later (“We expect high levels of heterogeneity”), you will have trouble performing a meta-analysis and providing narrative synthesis on all interventions because they might vary so much. I suggest that you:  o Formulate right from the beginning that you expect high levels of heterogeneity  o Therefore you will have subgroups (as you say later)  o And that you will try to answer your three research questions for each of the subgroup that you mention later (for example text messages interventions, apps, internet websites etc).  • Qualitative review :  Conducting a review on quantitative AND qualitative data is a very ambitious task. I understand you would like to provide a comprehensive view but I suggest some modifications about the qualitative data.  I have learned that one can perform a narrative review of qualitative studies or perform a systematic review (metasynthesis) where one goes beyond the results of the original studies and produces something that is more than their summary. If I am correct you will perform a narrative review for the qualitative part?  Furthermore, there’s a discrepancy, or something I don’t understand about what you name “qualitative outcomes”, reading those two sentences:  - “Previous reviews have focused mainly on quantitative HIV treatment outcomes and not on feasibility, acceptability, usability/functionality and perceived usefulness, which…”  - “Qualitative data on acceptability, feasibility, usability/functionality and perceived usefulness will be synthesized and reported narratively”  It is not really clear for me what is a qualitative outcome, and from my perspective qualitative data help us understand why an individual or a group think an intervention is useful, and therefore can help building future interventions. I would suggest not to select qualitative outcomes on feasibility, acceptability and so on but rather report patients’ perspectives on the intervention they are questioned about.  • There could be 3 questions for each subgroup:  o What exists?  o What works?  o Why does it work or not from adolescents’ perspective? => qualitative data  Of course it’s really simplified, but I leave it to you to see if it can fit the guidelines for systematic reviews and metaanalysis and I wish you good luck. | We thank the reviewer for the meaningful comments.  1 – the last 2 bullets were removed, and the section title changed to ‘conclusion.’  2 – we kept the recognised WHO definition in the introduction but indicated in the review that we will include an age range of up to 24 years. Thank you for the reference.  We removed exclusion criteria bullet 2.  3 – We added additional references.  4 - Thank you for the comment. We believe that the review questions are broad enough to include the options and sub-groups and prefer to keep the questions broad. It is implied that the questions will be asked per sub-group should the data permit it.  5 – We will not specifically perform a qualitative review/synthesis but indicated that descriptive and qualitative data will be analysed narratively/thematically. We have also addressed the confusion related to ‘qualitative outcomes’. Here we are merely referring to the secondary outcomes that are likely to be reported qualitatively.  Reference to ‘quantitative’ and ‘qualitative’ removed.  See our previous response. We believe that the current questions address the questions and mentioned by the reviewer and prefer to keep the questions broad and will apply them to the sub-groups should there be sufficient data. | 11  5  6  2  N/A  10  4, 5, 10  N/A |
