# Supplementary material for: Effectiveness, acceptability, and feasibility of technology-enabled health interventions for adolescents living with HIV in low- and middle-income countries: A systematic review protocol
Source: PLoS One. 2023 Feb 17;18(2):e0281894. doi: 10.1371/journal.pone.0281894 (PMC9937495; doi:10.1371/journal.pone.0281894)
Supplement: Attachment — Submitted filename: Response to Reviewers PLOS One_round 2.docx [file pone.0281894.s004.docx]

31 January 2023

PONE-D-22-18587

Effectiveness, acceptability and feasibility of technology-enabled health interventions for adolescents living with HIV in low- and middle-income countries: a systematic review protocol

Dear Editor,

We thank the reviewers for their time and effort in improving the manuscript. Please see our responses to the reviewers’ comments below.

| **Reviewer/Editor comment** | **Response** | **Page** |
| --- | --- | --- |
| Journal requirements  Please review your reference list to ensure that it is complete and correct. If you have cited papers that have been retracted, please include the rationale for doing so in the manuscript text, or remove these references and replace them with relevant current references. Any changes to the reference list should be mentioned in the rebuttal letter that accompanies your revised manuscript. If you need to cite a retracted article, indicate the article’s retracted status in the References list and also include a citation and full reference for the retraction notice. | We checked the reference list and added DOI numbers for all the studies that were applicable. There were no retracted articles. | 15-17 |
| Reviewer #1: Dear authors,  This is a very well-written protocol study, and now all my concerns are fulfilled.  Thanks for addressing all comments. | Thank you. | N/A |
| Reviewer #2: Dear Author,  Thank you for addressing all my comments. Well done as the manuscript has improved.  I still have one minor issue regarding the first two sentences of paragraph line 278 when you report your method for qualitative studies as I had troubles understanding them at first:  “Data on acceptability, feasibility, usability/functionality and perceived usefulness will be synthesised and reported narratively. Thematic qualitative analysis will be used to extract codes, categories and themes from the data related to acceptability, feasibility, usability/functionality and perceived usefulness.”  I suggest changing this in this way, if I am correct and if it does apply to what you intend to do:  “Data on acceptability, feasibility, usability/functionality and perceived usefulness from qualitative studies will be explored using thematic analysis and reported narratively.  Good luck with the review and its publication. | Thank you for the comment. We have accepted the suggestion of the reviewer. | 11 |
